# The Role of MicroRNAs in Arrhythmogenic Cardiomyopathy: Biomarkers or Innocent Bystanders of Disease Progression?

**DOI:** 10.3390/ijms21176434

**Published:** 2020-09-03

**Authors:** Maria Bueno Marinas, Rudy Celeghin, Marco Cason, Gaetano Thiene, Cristina Basso, Kalliopi Pilichou

**Affiliations:** Cardiovascular Pathology Unit, Department of Cardiac-Thoracic-Vascular Sciences and Public Health, University of Padua, 35121 Padua, Italy; maria.buenomarinas@unipd.it (M.B.M.); rudy.celeghin@gmail.com (R.C.); marco.cason@unipd.it (M.C.); gaetano.thiene@unipd.it (G.T.); cristina.basso@unipd.it (C.B)

**Keywords:** arrhythmogenic cardiomyopathy, microRNA, biomarker, pathogenesis

## Abstract

Arrhythmogenic cardiomyopathy (AC) is an inherited cardiac disease characterized by a progressive fibro-fatty replacement of the working myocardium and by life-threatening arrhythmias and risk of sudden cardiac death. Pathogenic variants are identified in nearly 50% of affected patients mostly in genes encoding for desmosomal proteins. AC incomplete penetrance and phenotypic variability advocate that other factors than genetics may modulate the disease, such as microRNAs (miRNAs). MiRNAs are small noncoding RNAs with a primary role in gene expression regulation and network of cellular processes. The implication of miRNAs in AC pathogenesis and their role as biomarkers for early disease detection or differential diagnosis has been the objective of multiple studies employing diverse designs and methodologies to detect miRNAs and measure their expression levels. Here we summarize experiments, evidence, and flaws of the different studies and hitherto knowledge of the implication of miRNAs in AC pathogenesis and diagnosis.

## 1. Introduction

Arrhythmogenic cardiomyopathy (AC) is a clinically and genetically heterogeneous disorder of heart muscle that is associated with ventricular arrhythmias and an increased risk of sudden cardiac death, especially in the young and athletes [1,2]. The prevalence of AC in the population ranges from 1:2000 to 1:5000 and affects more frequently males than females. AC penetrance is age-related and it becomes clinically overt in the second/fourth decade of life with rare symptoms/signs of the disease before puberty or in the elderly [3,4].

According to the most recent classification of cardiomyopathies, AC is a non-ischemic structural cardiomyopathy [5] showing progressive myocardial dystrophy with fibro-fatty tissue replacement of the ventricular myocardium. In an end-stage phase, hearts with congestive heart failure (HF) present biventricular involvement with multiple aneurysms and huge chamber dilatation [3]. However, hearts in the early phase of the disease have been described as normal in AC subjects and only a careful histological analysis may reveal subtle features such as left ventricular involvement confined to the posterolateral subepicardium [6].

### 1.1. AC Pathogenesis

The replacement of the working myocardium by fibro-fatty tissue is the hallmark of AC, however the specific mechanism by which the mechanical and/or functional disruption of cell junctions by mutant desmosomal proteins leads to cardiomyocyte death and subsequent repair with fibrous and fatty tissue is still under investigation [7]. Many theories have been proposed and are unlikely to be mutually exclusive. Even before the discovery of desmosomal genes in AC, electron microscopy studies demonstrated intercalated disc remodeling raising the hypothesis of an abnormal cell–cell adhesion in disease pathogenesis. Soon after, the role of intracellular signaling, first with the Wnt signaling pathway suppression leading to adipogenesis in a Dsp-deficient mouse model and next the Hippo/YAP signaling pathway, have been associated with AC pathogenesis [8,9,10,11].

### 1.2. AC Diagnosis 

Since the first comprehensive clinical description in 1982 [12] in which patients were described with ventricular tachyarrhythmias of left bundle branch block morphology, numerous criteria have been proposed to reach a reliable diagnosis, but no single criterion is sufficiently specific enough. In 1994, an international task force proposed to standardize the clinical diagnosis through a qualitative scoring system with major and minor criteria [13], which were revised extensively in 2010 to improve sensitivity (Table 1) [14]. Currently, AC diagnosis is multiparametric, combining multiple sources such as electrocardiographic, arrhythmic, morphofunctional, histopathological findings, and genetics [15,16].

### 1.3. AC Genetics

AC is considered a genetically determined disease caused by heterozygous or compound heterozygous mutations in genes mostly encoding proteins of the desmosomal complex in 50% of cases, with incomplete penetrance and variable expressivity, and mostly with an autosomic-dominant trait of inheritance [17]. The first disease-causing gene associated with AC was plakoglobin (*JUP*), identified in a fully penetrant autosomal-recessive form of AC associated with palmoplantar keratosis, also known as Naxos syndrome [18]. Consequently, desmoplakin (*DSP*) was associated with another autosomal recessive cardiocutaneous syndrome called Carvajal syndrome [19]. Soon after, numerous heterozygous mutations in *DSP* were identified in a dominant form of AC with/without skin abnormalities [20]. Nowadays, it is known that disease-causing mutations are detected in genes mostly encoding for proteins of the intercalated disc; being the most common mutant gene the plakophilin 2 gene (*PKP2*) found in 10–45% of cases, followed by *DSP* (10–15%), desmoglein 2 gene (*DSG2*) in 7–10% and finally desmocollin 2 (*DSC2*) in 2% [17]. Additionally, copy number variations in desmosomal genes have increased the diagnostic yield of genetic testing in AC [21]. Only isolated reports describe causal mutations in non-desmosomal genes, either linked to specific geographic regions or to phenotypic features—i.e., transmembrane protein 43 (*TMEM43*), desmin (*DES*), titin (*TTN*), lamin A/C (*LMNA*), phospholamban (*PLN*), αT-catenin (*CTNNA3*), ryanodine receptor 2 (*RYR2*), transforming growth factor β3 (*TGFβ3*), filamin C (*FLNC*), tight junction protein 1 (*TJP1*), and cadherin 2 (*CDH2*) (Table 2) [22]. Overall, genotyping success rate in AC reaches up to 50% based on cohort geographic localization and ethnicity, patients’ clinical criteria selection (definite/borderline/possible), the choice of sequencing techniques and the stringency of the standards by which mutations are considered causal. Adding also the fact that causal mutations are often low penetrance in AC leads to the assumption that the inheritance pattern of AC is more complex than previously appreciated, with frequent requirement for more than one ‘hit’ for fully penetrant disease or the need of other factors than genetics.

### 1.4. AC Biomarkers

The importance of biomarkers in the diagnosis and prognosis of AC is studied on several fronts, as they provide insight into the underlying pathological mechanisms and also help in guiding better management of disease. Imaging tools other than the echocardiography have been assessed showing that cardiac magnetic resonance (CMR) with the use of contrast means (i.e., gadolinium) provides a higher specificity in terms of non-invasive tissue characterization quantifying the presence and amount of fibrofatty myocardial scarring [44,45,46]. Initial diffidence regarding tissue characterization by CMR due to significant interobserver variability in the interpretation of segmental contraction analysis of the right ventricle (RV) free wall and low sensitivity/specificity, was overcome by recent studies which have demonstrated its great utility when combined wall motion alterations and pre-/post-contrast signal abnormalities. Circulating blood markers of HF have been postulated as AC biomarkers to ameliorate AC diagnosis since HF is often the end-stage of the disease. Circulating brain natriuretic peptide (BNP) has been demonstrated to be a useful indicator of RV dysfunction in AC patients, with a negative correlation with RV ejection fraction [47] as well as N-terminal fragment of BNP (NT-proBNP) [48]. Troponin I (cTnI), a well-known marker of cardiac injury, has been associated with AC, since it was demonstrated its correlation in pediatric patients with pathogenic disease-causing mutations showing evidence of myocardial inflammation at CMR [49]. However, the low specificity of these molecules due to their association to a large HF-related cardiac conditions limit their use as AC biomarkers. Likewise, chaperone heat shock protein 70 (HSP70) was proposed to be originated from damage myocardial cells in AC failing hearts [50] but it lacks specificity because of its high levels found also in dilated and ischemic cardiomyopathies [51]. Another circulating marker is bridging integrator 1 (BIN1) which was associated with ventricular arrhythmias in AC patients with HF [52] but lacked specificity [53]. Finally, interleukin-33 receptor ST2 associated with cardiac remodeling and fibrosis, has been correlated also to right/left ventricular dysfunction and arrhythmia occurrence [54] while Galectin-3 (GAL3) multifaceted inflammatory protein was found to be increased in plasma samples of AC patients adding a predictive value for ventricular arrhythmias (VA) [55].

The present review focuses on the recent studies on the role of miRNAs in AC in disease progression and gene regulation, and as potential biomarkers. MiRNA profiling studies conducted between 2016 and 2020 identified a spectrum of AC-related miRNAs using small/ large cohorts of patients, cell cultures or animal models. Herein, these profiled and validated miRNAs are classified according to the biological sample ie, whole blood, plasma, serum, cell cultures or cardiac muscle.

### 1.5. MicroRNAs

**Figure 1:**
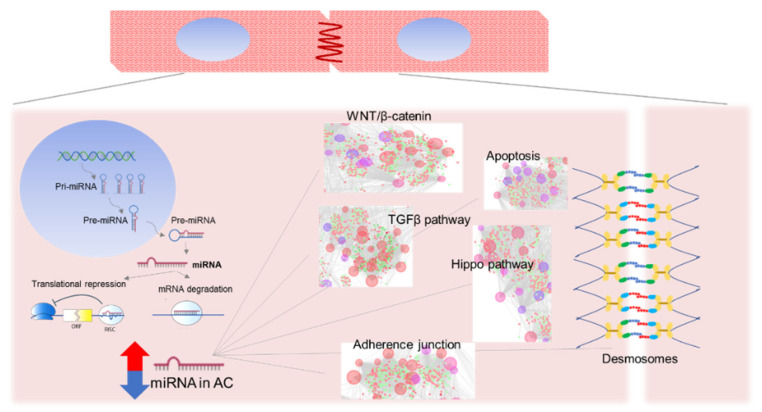
Schematic representation of miRNA maturation process, function and involvement in AC-related pathways.

MiRNAs maturation starts from a first transcribed molecule into the nucleus (pri-miRNA) which is processed in the cytoplasm into the effective and mature short miRNA of about 22 nucleotides (nt) [56]. The key function of this class of noncoding RNAs is gene-expression regulation by binding a specific messenger RNA (mRNA) leading to the breakdown of mRNAs and the inhibition of its subsequent translation into protein [57]. MiRNAs regulate gene expression by base pairing at sites in 3′ UTR of target mRNA. Binding specificity is only represented by 6 to 7 nucleotides from the above 20 of the mature miRNA, located between 2 to 8 nucleotides of 5′ end, calling this region the “seed region” [58] (Figure 1).

About 2000 miRNAs are coded by the human genome, which are grouped into families due to identical seed regions targeting similar groups of transcripts. Each miRNA family targets up to 300 mRNA targets [57]. MiRNA genes can be found in the intergenic regions either in isolation or in clusters or within exonic regions. Those in the intronic regions are under the control of their own promoters, whereas exonic miRNA genes overlap exon and intronic regions and either may be co-transcribed with the host gene or may also have their own promoters.

MiRNAs localization is either limited within the cell due to their main function targeting mRNAs and regulating protein synthesis, or extracellularly and in circulation acting as molecules mediating cell–cell communication. MiRNAs are present in all blood compartments, including plasma, platelets, erythrocytes, and nucleated blood cells [59]. In fact miRNAs have a well-established role also in cell-to-cell communication [60,61] since their stability and resistance to high/low temperature, pH changes, and multiple freeze/thaw cycles, is strictly associated with their packaging in microvesicles, exosomes, or apoptotic bodies in circulation. Circulating miRNAs can be released from apoptotic or necrotic cells during cell death process as free miRNAs which may aggregate to RNA-binding proteins or miRNA limited in apoptotic bodies. They can also be released by active secretion cells acting as signals for cell-to-cell communication within exosomes or vesicles [62]. Different studies on atherosclerosis have demonstrated miRNAs ability to be packed into microvesicles or apoptotic bodies and actively secreted in human blood cells or in circulation, repectively. In addition, cells viral infected can also produce secreted exosomes containing specific miRNAs inside.

Altered expression levels of a large miRNAs spectrum have been implicated in cardiac remodeling, hypertrophy, fibrosis, apoptosis, hypoxia, cardiomyopathy, and heart failure and generally in the regulation of cardiovascular function. MiRNAs’ features make these biomolecules promising biomarkers and even potential therapeutic targets [60].

## 2. MicroRNAs in AC Human Samples

The first miRNA profiling was carried out in 2016 in 24 AC cases. Zhang et al., studied 1078 human miRNAs by using tissue from the right ventricle of end-stage AC patients and from 24 controls (ctrl). For analysis purposes, the 1078 miRNAs were separated in 154 groups (7 miRNAs and SNORD44) and analyzed by ‘S-poly(T) plus’ assay comparing AC versus ctrl samples. Twenty-three of the 1078 miRNAs differentially expressed (DE) in AC samples with at least 1.5-fold-change were further validated on each sample separately, and ROC curve analysis showed AUC values above 0.7. As Wnt and Hippo pathways are correlated with AC pathogenesis, the authors arbitrarily selected two miRNAs involved in these pathways, miR-21-5p and miR-135b, and searched for their potential targets by Gene Ontology (GO) and KEGG analyses in order to build a potential network of interaction. Two targets emerged: bone morphogenetic protein receptor type 2 (*BMPR2*), associated with adipogenesis, and Transforming Growth Factor Beta Receptor 2 (*TGFBR2*), related to extracellular matrix production and fibrosis [63]. Although this first approach using human specimens was remarkable, the lack of a validation cohort and the arbitrary choice of miRNAs in presence of others with higher DE or AUC is controversial.

Sommariva et al., identified miR-320a DE in plasma samples of 36 AC patients. The authors used plasma samples from 3 AC and 3 ctrl to screen 377 miRNAs, identifying 5 DE miRNAs which were further validated in the entire study cohort (36 AC, 53 ctrl, and 21 idiopathic ventricular tachycardia (IVT) patients-/). Only miR-320a showed statistically significant DE, but with a questionable fold-change value of 0.53. Subsequently, the authors assessed whether miR-320a levels were influenced upon lifestyle by separating the 36 AC patients in athletes (*n* = 13) and non-athletes (*n* = 23). Although the authors concluded that lifestyle does not modify miR-320a levels, a significant DE was demonstrated when AC group was compared to IVT, with a fold-change value of 0.78 and AUC of 0.69 [64]. Validation of miR-320a levels in a larger cohort might reinforce statistical significance and clarify its potential as non-invasive biomarker for AC.

Similarly, Yamada et al., carried out a pilot study on 6 plasma samples of AC patients and 6 ctrl to identify miRNA expression levels in AC patients with ventricular arrhythmias (VA). The authors first screened an 84-miRNA cardiac-correlated array identifying 17 DE miRNAs in AC patients, of which 11 candidate miRNAs presented 4 times higher or lower levels compared to ctrl. The 6 miRNAs upregulated and those 4 downregulated were subsequently validated in a larger cohort of 28 AC patients with definite diagnosis according to TFC, 11 borderline or possible, 23 IVT, and 33 ctrl, demonstrating that AC patients with definite diagnosis presented higher levels of miR-144-3p, miR-145-5p, miR-185-5p, and miR-494 compared to all other groups (with AUC values above 0.6). Among these miRNAs, the highest difference was observed in miR-144-3p, miR-145-5p, and miR-494, as these four miRNAs were downregulated in borderline/possible AC and IVT patients and upregulated in AC with definite diagnosis. Subsequent evaluation of these 4 miRNAs in 25 AC definite patients after catheter ablation and among them, 8 with recurrent VA, showed that miR-494 is the only miRNA with significantly higher levels in recurrent VA and AUC value of 0.83. MiR-494 was further transfected in H9c2 cells showing a higher activation of caspase 3, and the apoptotic pathway [65]. The authors linked, for the first time, plasmatic miRNA levels of definite/borderline AC patients with recurrent/non-recurrent VA in AC, even though a larger cohort is needed to determine their actual potential as AC biomarkers.

More recently, our group intersected a RV myocardial tissue to a blood miRNA profile in AC index genotype-positive cases and validated its results to other cardiovascular diseases cohorts in order to identify a reliable disease biomarker. At first, right-ventricle myocardial samples from 9 heart-transplanted AC patients and blood samples from 9 AC patients with a definite diagnosis underwent full screening for an 84-miRNA cardiac-related array and small RNA-Seq. A total of 10 DE miRNAs were identified in common in tissue and blood samples of AC patients with pathogenic mutations in AC-related genes. These 10 miRNAs were subsequently validated in a larger AC cohort comprising 90 AC index cases with/without pathogenic mutations in AC-related genes (gen+/gen−), reducing significantly DE miRNAs to 6: miR-122-5p, miR-142-3p, miR-182-5p, miR-183-5p, miR-133a-3p, and miR-133b. This 6-miRNA panel was then assessed in five independent validation cohorts: patients affected by hypertrophic cardiomyopathy (HCM), dilated cardiomyopathy (DCM), Brugada Syndrome (BrS) myocarditis, and AC unaffected family members carrying a pathogenic mutation (gen + phen−). Even though each group showed some of these miRNAs DE, only AC definite patients shared this unique 6-miRNA profile (AUC 0.995 AC definite vs. ctrl; >0.82 AC definite vs. validation cohorts) which support its role as non-invasive biomarker. *In silico* predictions showed a clear correlation of this 6-miRNA profile to AC-related signaling pathways but functional studies are needed to determine its role in disease pathogenesis [66].

Only four studies hitherto were focused on miRNA expression evaluation in samples from AC affected patients (tissue, plasma, blood) demonstrating the promising role of these tiny molecules as non-invasive biomarkers essential in the clinical practice to distinguish disease phenocopies (Table 3). Incongruences among these studies might have occurred due to the use of different specimens and methods for miRNA quantification, thus larger patient cohorts and standardized methodologies are necessary to identify a univocal biomarker with high sensitivity and specificity.

## 3. MiRNAs in Cell Culture and AC Animal Models

Experiments in cell culture and animal models mimicking AC phenotype have tried to correlate miRNAs with AC pathogenesis (Table 4).

In 2016, Gurha et al., in an effort to determine a direct link between an AC-related gene and miRNA expression changes, performed a miRNA screening on *PKP2*-knockdown HL-1 cell culture (HL-1^Pkp2-shRNA^), identifying 59 DE miRNAs. Among these 59, miRNAs the most up/downregulated were miR-200b, miR-487b, miR-429, miR-184, miR-881, and miR-184. The expression of miR-184 was further evaluated in the heart tissue of two different mouse models, a *Pkp2* silenced specifically in the heart (*Nkx2.5-Cre:Pkp2^s^*^hRNA^) and the well-established *Jup* transgenic (*Myh6:Jup*^Tr^) mouse models [73], showing a significant reduction of miR-184 levels at early age (4-week-old mice).

Furthermore, a gene expression study in HL-1^Pkp2-shRNA^ cells showed alteration of Wnt, Hippo, and integrin pathways, but no direct link between these signaling pathways and miR-184, instead it was demonstrated a downregulation of E2F1 targets directly from miR-184. Finally, suppressed expression of miR-184 in both HL-1 and MPC cells was linked to a transcriptional switch to adipogenesis and enhancement of fat accumulation in vitro, as miR-184 directly targeted adipogenic genes [67]. Even though, through a complex series of experiments, the authors managed to link PKP2 deficiency to decreased miR-184 expression levels, *Myh6:Jup*^Tr^ mice also showed reduction of miR-184 expression level. As such, whether miR-184 expression level is linked to the lack of PKP2 or is correlated to changes in the entire desmosomal complex needs to be further elucidated.

A year later, Mazurek et al., described a transgenic mouse model with the ability to overexpress miR-130a specifically in the heart in the absence of doxycycline (αMHC-miR130a). This was the first AC model implicating miRNAs as potential causes of desmosomal dysfunction. αMHC-miR130a mice displayed left ventricular dysfunction (after 12 weeks), significantly increased right ventricular volumes but no differences in wall thickness (at 10–12 weeks), presence of premature ventricular complexes (at 8–10 weeks); and typical features of AC phenotype such as spotty fibrosis, lipid accumulation, and increased cell death (at 10 weeks). Bioinformatic analysis of the 3′ UTR regions in major desmosomal and junction proteins demonstrated that DSC2 was directly targeted by miR-130a, and DSC2 decreased protein levels were found in the myocardial tissue of αMHC-miR130a mice. Finally, in vitro target assay by luciferase reporter demonstrated that miR-130a acts directly as a translational repressor of DSC2, so its overexpression may influence in the dysfunction of the intercalated discs. The authors concluded that miR-130a overexpression leads to a disease phenotype resembling AC and may be used as potential model for miRNA-induced AC [68]. Even though miR-130a overexpression may lead to an AC phenotype, no miR-130a overexpression is found in human AC specimens or experimental models with the disease phenotype.

Recently, Calore et al. described a transgenic mouse with cardiomyocyte-specific overexpression of the human WT DSG2 (Tg-hWT) and p.Q558X DSG2 mutation (Tg-hQ). Histological and ultrastructural examinations showed in Tg-hQ loss of cardiomyocytes, signs of fibrosis (at 12 months), and lower number of desmosomes (at 6 months), respectively. Wnt/β-catenin signaling was also assessed, showing a reduction of ABC in Tg-hQ mice (at 3 and 6 months), indicating a significant change of β-catenin regulation since early disease stages. MiRNA analysis was performed on 3 Tg-hQ and 3 wild-type mice (at 6 months) showing 23 miRNAs altered, 18 upregulated and 6 downregulated. Of them, 3 miRNAs were selected and further validated by comparing Tg-hQ to Tg-hWT,: miR-499-5p, miR-217-5p, and miR-708-5p. A deep *in silico* analysis associated these 3 miRNAs with the regulation of Wnt/β-catenin signaling, adherence junction and gap junction [69], but no experimental evidence has been showed. As stated also from the authors, miRNA study in different AC models did not produce the same results probably due to different genetic background or differences in technique used. The forced overexpression of a truncating DSG2 produces a mutant human desmoglein-2 protein, which can functionally be compensated from the mouse endogenous desmoglein-2. As such it is not clear whether the AC phenotype observed in this mouse model as well as miRNAs DE are the result of the aberrant overexpression of the transgene or other mutations introduced with the human protein.

Cardiac stromal cells (CStCs) are an abundant cell population with differentiation potency and recently characterized as source of adipocytes in AC [70]. In this setting, cell cultures of CStCs were obtained from endomyocardial biopsies of 8 AC patients with a definite diagnosis and 7 ctrl. At first, whole genome miRNA expression profiling was performed on RNA from CStCs of 3 AC samples and 3 ctrl, identifying miR-520c-3p, miR-29b-3p, and miR-1183. Subsequently these miRNAs were validated in a total of 8 AC samples and 5 ctrl, confirming only miR-29b-3p. *In silico* analysis showed that miR-29b-3p targets genes involved in the extracellular matrix organization. Parallel gene expression analysis on CStCs derived from 7 AC and 7 ctrl, identified *SAXO2* and *NEDD9* as DE genes. Combining the miRNA and gene expression data set, the authors showed that a downregulation of target genes of the identified AC-specific miRNAs can be detected on RNA levels and they carried out a gene network analysis, which showed that DE genes are involved to cell-adhesion related processes, lipid transport, inflammation, and fibrosis related processes [71].

MiRNAs and mitochondrial DNA (mtDNA) expression analysis were also carried out in the conditioned medium collected from cardiomyocytes differentiated from induced pluripotent stem cells (iPSCs) of an AC patient and a ctrl. On day 30 of differentiation, cells demonstrated typical features of cardiomyocytes such as spontaneous contractions, generation of striated sarcomere and cardiomyocyte-specific miRNA expression. Eight extracellular miRNAs were assessed in the precipitated fractions after centrifugation and in the supernatant fractions. Of them, miR-21, miR-29b-2 and miR-378a expression levels were increased in the supernatant fraction of AC-derived cells compared to ctrl, whereas miR-1 and miR-133a expression levels were higher both in the supernatant and precipitated fractions in AC vs. ctrl. Subsequent analysis of intracellular miRNAs showed no DE miRNAs when comparing AC-derived IPSCs to ctrl, suggesting that differences appear upon secretion and not inside the cells. No differences were found in mtDNA content between supernatant and precipitated fractions, even though mtDNA content was significantly lower in supernatant fraction of AC-derived IPSCs compared to ctrl, suggesting that there is no association with inflammation [74].

Last, Puzzi et al., studied the biomechanical properties of HL-1 knockdown PKP2 cells through atomic force microscopy (AFM), showing that PKP2 deficiency leads to a decrease in cellular stiffness and perturbance of the actin network. Gene expression analysis and miR200 family levels (miR-200a-3p, miR-200b-3p and miR-429-3p) evaluation, demonstrated a downregulation of focal adhesion pathway and an upregulation of all three members of miR200 family. Subsequent search of possible miR200 targets resulted in seven candidate genes, among which *Col4a4*, *Col4a5*, *Itga1*, and *Src* which were significantly downregulated. AntagomiR, anti-miR-200b, was used to rescue Itga1 expression levels, and partially the mechanical properties of the cells, exception made for the plasticity index. The use of anti-miR-200b managed to recover completely cell-collagen adhesion, demonstrating the important role of miR-200b in the mechanical properties of PKP2-deficient cells. This complex experimental design with the use of AFM demonstrated a link between PKP2 and ITGA1 through the upregulation of miR-200b providing insights into the lack of adhesion and the altered biochemical properties in PKP2-defciency cells [72].

Taken altogether these experiments (Table 4) which are driven by the common hypothesis of understanding the role of miRNAs in AC pathogenesis, we now appreciate that miRNA-gene expression regulation is more complex and intriguing than expected. Differences in data analysis, statistical methods, and experimental conditions (cell culture, animal models, relative quantification, immunohistochemical evaluation, etc.) make it even harder to numerically compare the impact and the effect of each miRNA. Even though it is not stated in the reported studies, all miRNAs from animal models and cell-culture experiments are highly conserved among species according to current *in silico* tools, but this does not exclude functional differences of these miRNAs among species since cardiac physiology is different [75].

## 4. Conclusions

MiRNAs have a well-established role in different biological processes, normal or pathological, but the specific mechanism by which these molecules take part of a full and complex pathological process is intriguing. In addition, their high stability in biological samples and their presence also in circulation reflecting the changes occurring inside cells suggest a potential role as possible biomarkers. In this review, we summarized the hitherto knowledge of miRNAs specifically in AC.

An increasing number of studies world-wide on AC human samples, cell cultures or animal models mimicking AC phenotype share the common belief that these molecules are potentially therapeutics as well as prognostic and diagnostic biomarkers for AC. Even though there was little consensus among these studies regarding the miRNA signatures, there is strong evidence that miRNAs play an important role in the disease pathogenesis as most of the miRNAs identified in these studies are correlated in well-established AC pathways such as Wnt/β-catenin or Hippo pathways. Different techniques, specimens, and analysis methods might be a plausible explanation for the lack of reproducible associations.

The variable expressivity in AC patients carrying pathogenic mutations, the lack of genetic causes in about half of the affected patients and the lack of a gold-standard criterion for clinical diagnosis, highlight the need to dig deeper into the use of miRNAs as biomarkers in the clinical setting or their role in disease manifestation. To this regard, a joint effort to analyze results, compare methodologies, formally test the robustness of miRNA associations, and ultimately move towards to validate miRNA biomarkers is required.

## Figures and Tables

**Table 1:** 2010 Revised Task Force Criteria for AC

I. Global or Regional Dysfunction and Structural Alterations *	
**Major**	**Minor**
By 2D echo	By 2D echo
Regional RV akinesia, dyskinesia, or aneurysm and 1 of the following (end diastole):	Regional RV akinesia or dyskinesia and 1 of the following (end diastole):
PLAX RVOT ≥ 32 mm (corrected for body size (LAX/BSA) ≥ 19 mm/m^2^) PSAX RVOT ≥ 36 mm (corrected for body size (PSAX/BSA) ≥ 21 mm/m^2^) or fractional area change ≤ 33%	PLAX RVOT ≥ 29 to < 32 mm (corrected for body size (PLAX/BSA) ≥ 16 to < 19 m/m^2^) PSAX RVOT ≥ 32 to < 36 mm (corrected for body size (PSAX/BSA) ≥ 18 to < 21 mm/m^2^) or fractional area change > 33% to ≤40%
By CMR	By CMR
Regional RV akinesia or dyskinesia or dyssynchronous RV contraction and 1 of the following:	Regional RV akinesia or dyskinesia or dyssynchronous RV contraction and 1 of the following:
Ratio of RV end-diastolic volume to BSA ≥110 mL/m^2^ (male) or ≥ 100 mL/m^2^ (female) or RV ejection fraction ≤ 40%	Ratio of RV end-diastolic volume to BSA ≥ 100 to <110 mL/m^2^ (male) or ≥ 90 to < 100 mL/m^2^ (female) or RV ejection fraction > 40% to ≤ 45%
By RV angiography	
Regional RV akinesia, dyskinesia, or aneurysm	
**II. Tissue Characterization of Wall**	
**Major**	**Minor**
Residual myocytes <60% by morphometric analysis (or <50% if estimated), with fibrous replacement of the RV free wall myocardium in ≥1 sample, with or without fatty replacement of tissue on EMB	Residual myocytes <60% by morphometric analysis (or <50% if estimated), with fibrous replacement of the RV free wall myocardium in ≥1 sample, with or without fatty replacement of tissue on EMB
**III. Repolarization Abnormalities**	
**Major**	**Minor**
Inverted T waves in right precordial leads (V1, V2, and V3) or beyond in individuals >14 years of age (in the absence of complete RBBB QRS ≥ 120 ms)	Inverted T waves in leads V1 and V2 in individuals >14 years of age (in the absence of complete RBBB) or in V4, V5, or V6 Inverted T waves in leads V1, V2, V3, and V4 in individuals >14 years of age in the presence of complete right RBBB
**IV. Depolarization/Conduction Abnormalities**	
**Major**	**Minor**
Epsilon wave (reproducible low-amplitude signals between end of QRS complex to onset of the T wave) in the right precordial leads (V1 to V3)	Late potentials by SAECG in ≥1 of 3 parameters in the absence of aQRS duration of ≥110 ms on the standard ECG Filtered QRS duration (fQRS) ≥ 114 ms Duration of terminal QRS < 40 μV (low-amplitude signal duration) ≥ 38 ms Root-mean-square voltage of terminal 40 ms ≤ 20 μV Terminal activation duration of QRS ≥ 55 ms measured from the nadir of the S wave to the end of the QRS, including R’, in V1, V2, or V3, in the absence of complete RBBB
**V. Arrhythmias**	
**Major**	**Minor**
Nonsustained or sustained VT of LBBB morphology with superior axis (negative or indeterminate QRS in leads II, III, and aVF and positive in lead aVL)	Nonsustained or sustained VT of RVOT configuration, LBBB morphology with inferior axis (positive QRS in leads II, III, and aVF and negative in lead aVL) or of unknown axis >500 PVCs per 24 h (Holter)
**VI. Family History**	
**Major**	**Minor**
AC confirmed in a first-degree relative who meets current task force criteria AC confirmed pathologically at autopsy or surgery in a first-degree relative Identification of a pathogenic mutation^†^ categorized as associated or probably associated with AC in the patient under evaluation	History of AC in a first-degree relative in whom it is not possible or practical to determine whether the family member meets current task force criteria Premature SD (35 years of age) due to suspected AC in a first-degree relative AC confirmed pathologically or by current task force criteria in second-degree relative

Two major, or one major and two minor, or four minor criteria: definite diagnosis of AC. One major and one minor, or three minor criteria: borderline diagnosis; One major, or two minor criteria from different categories: possible diagnosis * Hypokinesis is not included in this or subsequent definitions of RV regional wall motion abnormalities for the proposed modified criteria ^†^ A pathogenic mutation is a DNA alteration associated with AC that alters or is expected to alter the encoded protein, is unobserved or rare in a large non- AC control population, and either alters or is predicted to alter the structure or function of the protein or has demonstrated linkage to the disease phenotype in a conclusive pedigree Abbreviations. BSA: body surface area; CMR: cardiac magnetic resonance; EMB: endomyocardial biopsy; LBBB: left bundle- branch block; PLAX: parasternal long-axis view; PSAX: parasternal short-axis view; PVC: premature ventricular complex; RBBB: right bundle-branch block; RV: right ventricle; RVOT: RV outflow tract; SD: sudden death; VT: ventricular tachycardia.

**Table 2:** Genetic involved in AC

Locus	Disease gene	Gene	Mode of Transmission	Reference	Comment
Desmosomal genes					
17q21.2	Plakoglobin	JUP	AD/AR	Mckoy et al. [23]	AR form: cardiocutaneous syndrome
6p24.3	Desmoplakin	DSP	AD/AR	Rampazzo et al. [20]	AR form: cardiocutaneous syndrome
12p11.21	Plakophilin-2	PKP2	AD/AR	Gerull et al. [24]	
18q12.1	Desmoglein-2	DSG2	AD/AR	Pilichou et al. [25]	
18q12.1	Desmocollin-2	DSC2	AD/AR	Syrris et al. [26]	
Non-desmosomal genes					
1q43	Cardiac Ryanodine Receptor 2	RYR2	AD	Tiso et al. [27]	CPVT
14q24.3	Transforming growth factor-beta-3	TGFB3	AD	Beffagna et al. [28]	Debated
3p25.1	Transmembrane Protein 43	TMEM43	AD	Merner et al. [29]	Limited to the founder p.Ser358Leu variant leading to fully disease penetrance.
2q35	Desmin	DES	AD	Van Tintelen et al. [30]	Overlap syndrome (DCM and HCM phenotype, early conduction disease)
6q22.31	Phospholamban	PLN	AD	Van der Zwaag et al. [31]	Limited to the founder p.Arg14del variant.
2q31.2	Titin	TTN	AD	Taylor et al. [32]	Overlap syndrome (early conduction disease, AF)
1q22	Lamin A/C	LMNA	AD	Quarta et al. [33]	Overlap syndrome (DCM, early conduction disease)
14q11.2	Myosin heavy chain 7	MYH7		De Bortoli et al. [34]	Debated overlap HCM
				Murray et al. [35]	
3p21.31	Myosin light Chain 3	MYL3		Murray et al. [35]	Debated overlap HCM
11p11.2	Myosin binding protein C3	MYBPC3		Sakamoto et al. [36]	Debated overlap HCM
				Murray et al. [35]	
10q23.2	LIM domain binding 3	LDB3		Lopez-Ayala et al. [37]	Debated
3p22.2	Sodium voltage-gated channel alpha subunit 5	SCN5A		Te Riele et al. [38]	Debated overlap BrS
10q21.3	Alpha-T-catenin	CTNNA3	AD	Van Hengel et al. [39]	
18q12.1	Cadherin-2	CDH2	AD	Mayosi et al. [40]	
				Turkowski et al. [41]	
15q13.1	Tight junction protein 1	TJP1	AD	De Bortoli et al. [42]	
7q32.1	Filamin C	FLNC	AD	Ortiz-Genga et al. [43]	Overlap syndrome (DCM and adverse outcome)

AD: autosomal dominant; AF: atrial fibrillation; AR: autosomal recessive; CPVT: catecholaminergic polymorphic ventricular tachycardia; DCM: dilated cardiomyopathy; HCM: hypertrophic cardiomyopathy, BrS: Brugada Syndrome.

**Table 3:** Differentially expressed miRNAs in human samples

miRNA	Specimen	Differential Diagnosis	AUC	Pathways	Reference
miR-21-5p	RV myocardial tissue	AC vs. ctrl	0.944	Wnt and Hippo pathways	[63]
miR-135b	RV myocardial tissue	AC vs. ctrl	0.936		
miR-320a	Plasma	AC vs. ctrl	/	/	[64]
		AC vs. IVT	0.69		
miR-144-3p	Plasma	AC definite vs. crtl AC definite vs. AC borderline/possible AC definite vs. IVT	/	/	
miR-145-5p	Plasma			/	[65]
miR-185-5p	Plasma	AC recurrent VA vs. no VA	0.728 0.619 0.740 0.891		
miR-494	Plasma			Activation of caspase 3, apoptosis related	
miR-122-5p	RV myocardial tissue and blood	AC definite vs. ctrl	0.995	Adherence junction, Hippo pathway, TFGβ signaling pathway, AC pathway, Wnt/β-catenin pathway	
miR-142-3p	RV myocardial tissue and blood	AC definite vs. HCM	0.804		[66]
miR-182-5p	RV myocardial tissue and blood	AC definite vs. DCM	0.917		
miR-183-5p	RV myocardial tissue and blood	AC definite vs. AC gen + phen	0.825		
miR-133a-3p	RV myocardial tissue and blood	AC definite vs. BrS	0.981		
miR-133b	RV myocardial tissue and blood	AC definite vs. Myocarditis	0.978		

Summary of DE miRNAs identified in AC-human samples, specimens used, comparison groups analyzed, AUC values, pathways in which DE miRNAs are involved. RV: right-ventricle. AC: arrhythmogenic cardiomyopathy; ctrl: controls; IVT: idiopathic ventricular tachycardia; VA: ventricular arrhythmias; HCM: hypertrophic cardiomyopathy; DCM: dilated cardiomyopathy; AC gen + phen −: unaffected family members carriers of a pathogenic variant in AC-related gene; BrS: Brugada syndrome; AUC: area under the curve.

**Table 4:** Differentially expressed miRNAs in cell culture and animal models

miRNA	Specimen	Pathways	Reference
miR-184	HL-1^Pkp2-shRNA^	PKP2 deficiency → miR-184 dysregulation and Wnt, Hippo and integrin pathways E2F1 regulates directly miR-184 by epigenetic silencing	[67]
	Mouse Nkx2.5-Cre:Pkp2^shRNA^		
	Mouse Myh6:Jup^Tr^		
miR-130	Mouse αMHC-miR130a (overexpressing miR-130a)	Spontaneous onset of AC phenotype (LV dysfunction, fibrosis and lipid accumulation, increase myocyte death). DSC2 as predicted target.	[68]
miR-499-5p	Mouse Dsg2 Tg-hWT and Tg-hQ (transgenic WT and Dsg2 p.Q558X)	Loss of cardiomyocytes and fibrosis at 12 months Lower number of desmosomes at 6 months MiRNAs related with Wnt/β-catenin, adherence juntion and gap junction pathways	[69]
miR-217-5p			
miR-708-5p			
miR-29b-3p	CStCs from AC patients	Cell-adhesion, lipid transport, inflammation, and fibrosis related processes.	[70]
miR-21	Cardiomyocytes derived from iPSCs from AC patients	Dysregulation due to differences in secretion of miRNAs from cells.	[71]
miR-29b-2			
miR-378a			
miR-1			
miR-133a			
miR-200b	HL-1 PKP2 deficiency	PKP2 deficiency → miR-200b upregulation → Itga1 suppression Decrease on cellular stiffness and perturbance of actin network	[72]

Summary of DE miRNAs identified in cell culture and AC animal models, type of cell culture and animal model used and pathways involved. PKP2: plakophilin-2; Dsg2: desmoglein-2; Tg: transgenic; WT: wild-type; CStCs: cardiac stromal cells; iPSCs: induced pluripotent stem cells; Itga1: intengrin subunit alpha 1.

## References

[1] Thiene G., Nava A., Corrado D., Rossi L., Pennelli N. (1988). Right ventricular cardiomyopathy and sudden death in young people. N. Engl. J. Med..

[2] Corrado D., Thiene G., Nava A., Rossi L., Pennelli N. (1990). Sudden death in young competitive athletes: Clinicopathologic correlations in 22 cases. Am. J. Med..

[3] Basso C., Thiene G., Corrado D., Angelini A., Nava A., Valente M. (1996). Arrhythmogenic right ventricular cardiomyopathy. Dysplasia, dystrophy, or myocarditis?. Circulation.

[4] Nava A., Bauce B., Basso C., Muriago M., Rampazzo A., Villanova C., Daliento L., Buja G., Corrado D., Danieli G.A. (2000). Clinical profile and long-term follow-up of 37 families with arrhythmogenic right ventricular cardiomyopathy. J. Am. Coll. Cardiol..

[5] Maron B.J. (2008). The 2006 american heart association classification of cardiomyopathies is the gold standard. Circ. Heart Fail.

[6] Corrado D., Basso C., Thiene G., McKenna W.J., Davies M.J., Fontaliran F., Nava A., Silvestri F., Blomstrom-Lundqvist C., Wlodarska E.K. (1997). Spectrum of clinicopathologic manifestations of arrhythmogenic right ventricular cardiomyopathy/dysplasia: A multicenter study. J. Am. Coll. Cardiol..

[7] Basso C., Corrado D., Marcus F.I., Nava A., Thiene G. (2009). Arrhythmogenic right ventricular cardiomyopathy. Lancet.

[8] Garcia-Gras E., Lombardi R., Giocondo M.J., Willerson J.T., Schneider M.D., Khoury D.S., Marian A.J. (2006). Suppression of canonical wnt/beta-catenin signaling by nuclear plakoglobin recapitulates phenotype of arrhythmogenic right ventricular cardiomyopathy. J. Clin. Investig..

[9] Lombardi R., Dong J., Rodriguez G., Bell A., Leung T.K., Schwartz R.J., Willerson J.T., Brugada R., Marian A.J. (2009). Genetic fate mapping identifies second heart field progenitor cells as a source of adipocytes in arrhythmogenic right ventricular cardiomyopathy. Circ. Res..

[10] Chen S.N., Gurha P., Lombardi R., Ruggiero A., Willerson J.T., Marian A.J. (2014). The hippo pathway is activated and is a causal mechanism for adipogenesis in arrhythmogenic cardiomyopathy. Circ. Res..

[11] Basso C., Pilichou K., Bauce B., Corrado D., Thiene G. (2018). Diagnostic criteria, genetics, and molecular basis of arrhythmogenic cardiomyopathy. Heart Fail Clin..

[12] Marcus F.I., Fontaine G.H., Guiraudon G., Frank R., Laurenceau J.L., Malergue C., Grosgogeat Y. (1982). Right ventricular dysplasia: A report of 24 adult cases. Circulation.

[13] McKenna W.J., Thiene G., Nava A., Fontaliran F., Blomstrom-Lundqvist C., Fontaine G., Camerini F. (1994). Diagnosis of arrhythmogenic right ventricular dysplasia/cardiomyopathy. Task force of the working group myocardial and pericardial disease of the european society of cardiology and of the scientific council on cardiomyopathies of the international society and federation of cardiology. Br. Heart J..

[14] Marcus F.I., McKenna W.J., Sherrill D., Basso C., Bauce B., Bluemke D.A., Calkins H., Corrado D., Cox M.G., Daubert J.P. (2010). Diagnosis of arrhythmogenic right ventricular cardiomyopathy/dysplasia: Proposed modification of the task force criteria. Eur. Heart J..

[15] Corrado D., Perazzolo Marra M., Zorzi A., Beffagna G., Cipriani A., Lazzari M., Migliore F., Pilichou K., Rampazzo A., Rigato I. (2020). Diagnosis of arrhythmogenic cardiomyopathy: The padua criteria. Int. J. Cardiol..

[16] Corrado D., van Tintelen P.J., McKenna W.J., Hauer R.N.W., Anastastakis A., Asimaki A., Basso C., Bauce B., Brunckhorst C., Bucciarelli-Ducci C. (2020). Arrhythmogenic right ventricular cardiomyopathy: Evaluation of the current diagnostic criteria and differential diagnosis. Eur. Heart J..

[17] Pilichou K., Thiene G., Bauce B., Rigato I., Lazzarini E., Migliore F., Perazzolo Marra M., Rizzo S., Zorzi A., Daliento L. (2016). Arrhythmogenic cardiomyopathy. Orphanet J. Rare Dis..

[18] Protonotarios N., Tsatsopoulou A., Patsourakos P., Alexopoulos D., Gezerlis P., Simitsis S., Scampardonis G. (1986). Cardiac abnormalities in familial palmoplantar keratosis. Br. Heart J..

[19] Norgett E.E., Hatsell S.J., Carvajal-Huerta L., Cabezas J.C., Common J., Purkis P.E., Whittock N., Leigh I.M., Stevens H.P., Kelsell D.P. (2000). Recessive mutation in desmoplakin disrupts desmoplakin-intermediate filament interactions and causes dilated cardiomyopathy, woolly hair and keratoderma. Hum. Mol. Genet..

[20] Rampazzo A., Nava A., Malacrida S., Beffagna G., Bauce B., Rossi V., Zimbello R., Simionati B., Basso C., Thiene G. (2002). Mutation in human desmoplakin domain binding to plakoglobin causes a dominant form of arrhythmogenic right ventricular cardiomyopathy. Am. J. Hum. Genet..

[21] Pilichou K., Lazzarini E., Rigato I., Celeghin R., de Bortoli M., Perazzolo Marra M., Cason M., Jongbloed J., Calore M., Rizzo S. (2017). Large genomic rearrangements of desmosomal genes in italian arrhythmogenic cardiomyopathy patients. Circ. Arrhythm. Electrophysiol..

[22] Celeghin R., Thiene G., Bauce B., Basso C., Pilichou K. (2019). Genetics in cardiovascular diseases. Ital. J. Med..

[23] McKoy G., Protonotarios N., Crosby A., Tsatsopoulou A., Anastasakis A., Coonar A., Norman M., Baboonian C., Jeffery S., McKenna W.J. (2000). Identification of a deletion in plakoglobin in arrhythmogenic right ventricular cardiomyopathy with palmoplantar keratoderma and woolly hair (naxos disease). Lancet.

[24] Gerull B., Heuser A., Wichter T., Paul M., Basson C.T., McDermott D.A., Lerman B.B., Markowitz S.M., Ellinor P.T., MacRae C.A. (2004). Mutations in the desmosomal protein plakophilin-2 are common in arrhythmogenic right ventricular cardiomyopathy. Nat. Genet..

[25] Pilichou K., Nava A., Basso C., Beffagna G., Bauce B., Lorenzon A., Frigo G., Vettori A., Valente M., Towbin J. (2006). Mutations in desmoglein-2 gene are associated with arrhythmogenic right ventricular cardiomyopathy. Circulation.

[26] Syrris P., Ward D., Evans A., Asimaki A., Gandjbakhch E., Sen-Chowdhry S., McKenna W.J. (2006). Arrhythmogenic right ventricular dysplasia/cardiomyopathy associated with mutations in the desmosomal gene desmocollin-2. Am. J. Hum. Genet..

[27] Tiso N., Stephan D.A., Nava A., Bagattin A., Devaney J.M., Stanchi F., Larderet G., Brahmbhatt B., Brown K., Bauce B. (2001). Identification of mutations in the cardiac ryanodine receptor gene in families affected with arrhythmogenic right ventricular cardiomyopathy type 2 (arvd2). Hum. Mol. Genet..

[28] Beffagna G., Occhi G., Nava A., Vitiello L., Ditadi A., Basso C., Bauce B., Carraro G., Thiene G., Towbin J.A. (2005). Regulatory mutations in transforming growth factor-beta3 gene cause arrhythmogenic right ventricular cardiomyopathy type 1. Cardiovasc. Res..

[29] Merner N.D., Hodgkinson K.A., Haywood A.F., Connors S., French V.M., Drenckhahn J.D., Kupprion C., Ramadanova K., Thierfelder L., McKenna W. (2008). Arrhythmogenic right ventricular cardiomyopathy type 5 is a fully penetrant, lethal arrhythmic disorder caused by a missense mutation in the tmem43 gene. Am. J. Hum. Genet..

[30] Van Tintelen J.P., van Gelder I.C., Asimaki A., Suurmeijer A.J., Wiesfeld A.C., Jongbloed J.D., van den Wijngaard A., Kuks J.B., van Spaendonck-Zwarts K.Y., Notermans N. (2009). Severe cardiac phenotype with right ventricular predominance in a large cohort of patients with a single missense mutation in the des gene. Heart Rhythm..

[31] Van der Zwaag P.A., van Rijsingen I.A., Asimaki A., Jongbloed J.D., van Veldhuisen D.J., Wiesfeld A.C., Cox M.G., van Lochem L.T., de Boer R.A., Hofstra R.M. (2012). Phospholamban r14del mutation in patients diagnosed with dilated cardiomyopathy or arrhythmogenic right ventricular cardiomyopathy: Evidence supporting the concept of arrhythmogenic cardiomyopathy. Eur. J. Heart Fail.

[32] Taylor M., Graw S., Sinagra G., Barnes C., Slavov D., Brun F., Pinamonti B., Salcedo E.E., Sauer W., Pyxaras S. (2011). Genetic variation in titin in arrhythmogenic right ventricular cardiomyopathy-overlap syndromes. Circulation.

[33] Quarta G., Syrris P., Ashworth M., Jenkins S., Zuborne Alapi K., Morgan J., Muir A., Pantazis A., McKenna W.J., Elliott P.M. (2012). Mutations in the lamin a/c gene mimic arrhythmogenic right ventricular cardiomyopathy. Eur. Heart J..

[34] De Bortoli M., Calore C., Lorenzon A., Calore M., Poloni G., Mazzotti E., Rigato I., Marra M.P., Melacini P., Iliceto S. (2017). Co-inheritance of mutations associated with arrhythmogenic cardiomyopathy and hypertrophic cardiomyopathy. Eur. J. Human Genet. EJHG.

[35] Murray B., Hoorntje E.T., Te Riele A., Tichnell C., van der Heijden J.F., Tandri H., van den Berg M.P., Jongbloed J.D.H., Wilde A.A.M., Hauer R.N.W. (2018). Identification of sarcomeric variants in probands with a clinical diagnosis of arrhythmogenic right ventricular cardiomyopathy (arvc). J. Cardiovasc. Electrophysiol..

[36] Sakamoto N., Natori S., Hosoguchi S., Minoshima A., Noro T., Akasaka K., Sato N., Ohno S., Ikeda Y., Ishibashi-Ueda H. (2019). Left-dominant arrhythmogenic cardiomyopathy with heterozygous mutations in dsp and mybpc3. Circ. Cardiovasc. Imaging.

[37] Lopez-Ayala J.M., Ortiz-Genga M., Gomez-Milanes I., Lopez-Cuenca D., Ruiz-Espejo F., Sanchez-Munoz J.J., Oliva-Sandoval M.J., Monserrat L., Gimeno J.R. (2015). A mutation in the z-line cypher/zasp protein is associated with arrhythmogenic right ventricular cardiomyopathy. Clin. Genet..

[38] Te Riele A.S., Agullo-Pascual E., James C.A., Leo-Macias A., Cerrone M., Zhang M., Lin X., Lin B., Sobreira N.L., Amat-Alarcon N. (2017). Multilevel analyses of scn5a mutations in arrhythmogenic right ventricular dysplasia/cardiomyopathy suggest non-canonical mechanisms for disease pathogenesis. Cardiovasc. Res..

[39] Van Hengel J., Calore M., Bauce B., Dazzo E., Mazzotti E., de Bortoli M., Lorenzon A., Li Mura I.E., Beffagna G., Rigato I. (2013). Mutations in the area composita protein alphat-catenin are associated with arrhythmogenic right ventricular cardiomyopathy. Eur. Heart J..

[40] Mayosi B.M., Fish M., Shaboodien G., Mastantuono E., Kraus S., Wieland T., Kotta M.C., Chin A., Laing N., Ntusi N.B. (2017). Identification of cadherin 2 (cdh2) mutations in arrhythmogenic right ventricular cardiomyopathy. Circ. Cardiovasc. Genet..

[41] Turkowski K.L., Tester D.J., Bos J.M., Haugaa K.H., Ackerman M.J. (2017). Whole exome sequencing with genomic triangulation implicates cdh2-encoded n-cadherin as a novel pathogenic substrate for arrhythmogenic cardiomyopathy. Congenit. Heart Dis..

[42] De Bortoli M., Postma A.V., Poloni G., Calore M., Minervini G., Mazzotti E., Rigato I., Ebert M., Lorenzon A., Vazza G. (2018). Whole-exome sequencing identifies pathogenic variants in tjp1 gene associated with arrhythmogenic cardiomyopathy. Circ. Genom. Precis. Med..

[43] Ortiz-Genga M.F., Cuenca S., Dal Ferro M., Zorio E., Salgado-Aranda R., Climent V., Padron-Barthe L., Duro-Aguado I., Jimenez-Jaimez J., Hidalgo-Olivares V.M. (2016). Truncating flnc mutations are associated with high-risk dilated and arrhythmogenic cardiomyopathies. J. Am. Coll. Cardiol..

[44] Sen-Chowdhry S., McKenna W.J. (2008). The utility of magnetic resonance imaging in the evaluation of arrhythmogenic right ventricular cardiomyopathy. Curr. Opin. Cardiol..

[45] Corrado D., Basso C., Leoni L., Tokajuk B., Turrini P., Bauce B., Migliore F., Pavei A., Tarantini G., Napodano M. (2008). Three-dimensional electroanatomical voltage mapping and histologic evaluation of myocardial substrate in right ventricular outflow tract tachycardia. J. Am. Coll. Cardiol..

[46] Perazzolo Marra M., Rizzo S., Bauce B., de Lazzari M., Pilichou K., Corrado D., Thiene G., Iliceto S., Basso C. (2015). Arrhythmogenic right ventricular cardiomyopathy. Contribution of cardiac magnetic resonance imaging to the diagnosis. Herz.

[47] Matsuo K., Nishikimi T., Yutani C., Kurita T., Shimizu W., Taguchi A., Suyama K., Aihara N., Kamakura S., Kangawa K. (1998). Diagnostic value of plasma levels of brain natriuretic peptide in arrhythmogenic right ventricular dysplasia. Circulation.

[48] Cheng H., Lu M., Hou C., Chen X., Wang J., Yin G., Chu J., Zhang S., Prasad S.K., Pu J. (2015). Relation between n-terminal pro-brain natriuretic peptide and cardiac remodeling and function assessed by cardiovascular magnetic resonance imaging in patients with arrhythmogenic right ventricular cardiomyopathy. Am. J. Cardiol..

[49] Martins D., Ovaert C., Khraiche D., Boddaert N., Bonnet D., Raimondi F. (2018). Myocardial inflammation detected by cardiac mri in arrhythmogenic right ventricular cardiomyopathy: A paediatric case series. Int. J. Cardiol..

[50] Wei Y.J., Huang Y.X., Shen Y., Cui C.J., Zhang X.L., Zhang H., Hu S.S. (2009). Proteomic analysis reveals significant elevation of heat shock protein 70 in patients with chronic heart failure due to arrhythmogenic right ventricular cardiomyopathy. Mol. Cell Biochem..

[51] Stadiotti I., Pompilio G., Maione A.S., Pilato C.A., D’Alessandra Y., Sommariva E. (2019). Arrhythmogenic cardiomyopathy: What blood can reveal?. Heart Rhythm.

[52] Hong T.T., Cogswell R., James C.A., Kang G., Pullinger C.R., Malloy M.J., Kane J.P., Wojciak J., Calkins H., Scheinman M.M. (2012). Plasma bin1 correlates with heart failure and predicts arrhythmia in patients with arrhythmogenic right ventricular cardiomyopathy. Heart Rhythm.

[53] Asimaki A. (2012). Bin1: A new biomarker to track arvc?. Heart Rhythm.

[54] Broch K., Leren I.S., Saberniak J., Ueland T., Edvardsen T., Gullestad L., Haugaa K.H. (2017). Soluble st2 is associated with disease severity in arrhythmogenic right ventricular cardiomyopathy. Biomarkers.

[55] Oz F., Onur I., Elitok A., Ademoglu E., Altun I., Bilge A.K., Adalet K. (2017). Galectin-3 correlates with arrhythmogenic right ventricular cardiomyopathy and predicts the risk of ventricular -arrhythmias in patients with implantable defibrillators. Acta Cardiol..

[56] Ambros V. (2001). Micrornas: Tiny regulators with great potential. Cell.

[57] Bartel D.P. (2004). Micrornas: Genomics, biogenesis, mechanism, and function. Cell.

[58] Lai E.C. (2002). Micro rnas are complementary to 3’ utr sequence motifs that mediate negative post-transcriptional regulation. Nat. Genet..

[59] Latronico M.V., Condorelli G. (2009). Micrornas and cardiac pathology. Nat. Rev. Cardiol..

[60] Creemers E.E., Tijsen A.J., Pinto Y.M. (2012). Circulating micrornas: Novel biomarkers and extracellular communicators in cardiovascular disease?. Circ. Res..

[61] Winter J., Jung S., Keller S., Gregory R.I., Diederichs S. (2009). Many roads to maturity: Microrna biogenesis pathways and their regulation. Nat. Cell Biol..

[62] Schwarzenbach H., Nishida N., Calin G.A., Pantel K. (2014). Clinical relevance of circulating cell-free micrornas in cancer. Nat. Rev. Clin. Oncol..

[63] Zhang H., Liu S., Dong T., Yang J., Xie Y., Wu Y., Kang K., Hu S., Gou D., Wei Y. (2016). Profiling of differentially expressed micrornas in arrhythmogenic right ventricular cardiomyopathy. Sci. Rep..

[64] Sommariva E., D’Alessandra Y., Farina F.M., Casella M., Cattaneo F., Catto V., Chiesa M., Stadiotti I., Brambilla S., Dello Russo A. (2017). Mir-320a as a potential novel circulating biomarker of arrhythmogenic cardiomyopathy. Sci. Rep..

[65] Yamada S., Hsiao Y.W., Chang S.L., Lin Y.J., Lo L.W., Chung F.P., Chiang S.J., Hu Y.F., Tuan T.C., Chao T.F. (2018). Circulating micrornas in arrhythmogenic right ventricular cardiomyopathy with ventricular arrhythmia. Europace.

[66] Bueno Marinas M., Celeghin R., Cason M., Bariani R., Frigo A.C., Jager J., Syrris P., Elliott P.M., Bauce B., Thiene G. (2020). A microrna expression profile as non-invasive biomarker in a large arrhythmogenic cardiomyopathy cohort. Int. J. Mol. Sci..

[67] Gurha P., Chen X., Lombardi R., Willerson J.T., Marian A.J. (2016). Knockdown of plakophilin 2 downregulates mir-184 through cpg hypermethylation and suppression of the e2f1 pathway and leads to enhanced adipogenesis in vitro. Circ. Res..

[68] Mazurek S.R., Calway T., Harmon C., Farrell P., Kim G.H. (2017). Microrna-130a regulation of desmocollin 2 in a novel model of arrhythmogenic cardiomyopathy. Microrna.

[69] Calore M., Lorenzon A., Vitiello L., Poloni G., Khan M.A.F., Beffagna G., Dazzo E., Sacchetto C., Polishchuk R., Sabatelli P. (2019). A novel murine model for arrhythmogenic cardiomyopathy points to a pathogenic role of wnt signaling and mirna dysregulation. Cardiovasc. Res..

[70] Sommariva E., Brambilla S., Carbucicchio C., Gambini E., Meraviglia V., Dello Russo A., Farina F.M., Casella M., Catto V., Pontone G. (2016). Cardiac mesenchymal stromal cells are a source of adipocytes in arrhythmogenic cardiomyopathy. Eur. Heart J..

[71] Rainer J., Meraviglia V., Blankenburg H., Piubelli C., Pramstaller P.P., Paolin A., Cogliati E., Pompilio G., Sommariva E., Domingues F.S. (2018). The arrhythmogenic cardiomyopathy-specific coding and non-coding transcriptome in human cardiac stromal cells. BMC Genom..

[72] Puzzi L., Borin D., Gurha P., Lombardi R., Martinelli V., Weiss M., Andolfi L., Lazzarino M., Mestroni L., Marian A.J. (2019). Knock down of plakophillin 2 dysregulates adhesion pathway through upregulation of mir200b and alters the mechanical properties in cardiac cells. Cells.

[73] Lombardi R., da Graca Cabreira-Hansen M., Bell A., Fromm R.R., Willerson J.T., Marian A.J. (2011). Nuclear plakoglobin is essential for differentiation of cardiac progenitor cells to adipocytes in arrhythmogenic right ventricular cardiomyopathy. Circ. Res..

[74] Khudiakov A.A., Smolina N.A., Perepelina K.I., Malashicheva A.B., Kostareva A.A. (2019). Extracellular micrornas and mitochondrial DNA as potential biomarkers of arrhythmogenic cardiomyopathy. Biochem. Biokhimiia.

[75] Lindsey M.L., Kassiri Z., Virag J.A.I., de Castro Bras L.E., Scherrer-Crosbie M. (2018). Guidelines for measuring cardiac physiology in mice. Am. J. Physiol. Heart Circ. Physiol..

